# A Novel Image Classification Method Based on Residual Network, Inception, and Proposed Activation Function

**DOI:** 10.3390/s23062976

**Published:** 2023-03-09

**Authors:** Ali Abdullah Yahya, Kui Liu, Ammar Hawbani, Yibin Wang, Ali Naser Hadi

**Affiliations:** 1 School of Computer and Information, Anqing Normal University, Anqing 246011, China; 2 School of Computer and Technology, University of Science and Technology of China, Hefei 230027, China; 3 School of Computer and Information, Hefei University of Technology, Hefei 230009, China

**Keywords:** inception, non-monotonic activation function (NMAF), 1 × 1 convolutions, residual networks, symmetric factorization

## Abstract

In deeper layers, ResNet heavily depends on skip connections and Relu. Although skip connections have demonstrated their usefulness in networks, a major issue arises when the dimensions between layers are not consistent. In such cases, it is necessary to use techniques such as zero-padding or projection to match the dimensions between layers. These adjustments increase the complexity of the network architecture, resulting in an increase in parameter number and a rise in computational costs. Another problem is the vanishing gradient caused by utilizing Relu. In our model, after making appropriate adjustments to the inception blocks, we replace the deeper layers of ResNet with modified inception blocks and Relu with our non-monotonic activation function (NMAF). To reduce parameter number, we use symmetric factorization and 1×1 convolutions. Utilizing these two techniques contributed to reducing the parameter number by around 6 M parameters, which has helped reduce the run time by 30 s/epoch. Unlike Relu, NMAF addresses the deactivation problem of the non-positive number by activating the negative values and outputting small negative numbers instead of zero in Relu, which helped in enhancing the convergence speed and increasing the accuracy by 5%, 15%, and 5% for the non-noisy datasets, and 5%, 6%, 21% for non-noisy datasets.

## 1. Introduction

The recent trend to improve the classification accuracy of the neural network is to increase the number and size of layers [1].

Stacking further layers for learning better neural networks and getting higher classification accuracy is a way that always leads to exploding or vanishing gradients. This issue has been extensively addressed in the literature [2,3,4,5,6,7,8,9,10,11,12,13,14,15,16,17].

In [18], Peng et al. proposed a new approach that addresses the difficulties of training the deep neural network. The authors used the Inception-ResNet network to treat these difficulties. In this work, the authors proposed to initialize the value into small values to enhance the stability of the model training.

Min et al. [19] proposed a deep neural network called a network in the network. The authors suggested adding a nonlinear activation function after each 1×1 convolution to reduce the number of parameters and enhance computational efficiency.

Simonyan et al. addressed the impact of convolutional network depth on classification accuracy [20]. In this work, the authors proposed a new image classification model called VGG. In this model, 3×3 convolution filters are used to assess networks with increasing depth. The authors in this work have achieved considerable enhancements in classification ConvNet training [21]. The top-1 and top-5 errors are used to evaluate the classification performance.

Inspired by the shorter connections between layers, Huang et al. [22] proposed a novel deep neural network called a dense convolutional network (DenseNet). In this work, the authors used a feed-forward fashion to reduce the number of parameters and speed up the training process. DenseNets can enhance classification accuracy without significant performance penalties.

Victor et al. [23] proposed using pre-trained models such as ResNet-50 and VGG-19 to minimize the computing time and reduce the training data. In this study, the authors conducted a comparison between pre-trained models and the ones that are trained from scratch. Dropout regularization and data augmentation are used to reduce overfitting.

In [24], Cheng et al. proposed using a modular group attention block to extract the feature dependencies from medical images. In this approach, a new ResNet variant called ResGANet is created by accumulating the group attention blocks in the ResNet style. Experimental results demonstrated that ResGANet could reduce the number of parameters and improve medical image classification accuracy.

Sarwinda et al. [25] proposed applying the ResNet model to detect colorectal cancer. In this approach, ResNet-18 and ResNet-50 are trained on colon gland images to classify colorectal cancer into malignant and benign. Three dataset distribution models are built and used to evaluate the performance of the proposed model in terms of sensitivity, specificity values, and accuracy.

In [26],the dataset of interest is used to learn the model architecture. To reduce the cost of searching for the architectural building block, the authors proposed to search for the architectural building block in the small dataset, then transfer the block into a larger dataset. In this approach, a new search space called NASNet is adopted to make the transfer process as smooth as possible.

Zoph and Le [27] created neural network descriptions based on recurrent networks. In this work, a recurrent neural network is built and used to search in variable-length architecture space. For enhancing classification accuracy on a validation set, reinforcement learning is used to train the recurrent network.

Szegedy et al. [28,29,30] designed a deep convolutional neural network based on the 1×1 convolutions. The authors argued that applying 1×1 convolutions helps to reduce the computation time and the number of parameters, which allows for increasing the width and depth of the network without any severe performance penalty.

Clevert et al. [31] proposed a new activation function called exponential linear unit (ELU) to speed up the learning process of deep neural networks. Applying ELU helped to enhance learning characteristics and improve classification accuracy. In this aspect, the negative values in ELU were used to minimize the variation of the forward propagation and accelerate the learning process.

Chen et al. [32] proposed using a convolutional neural network to classify the hyperspectral image. In this work, the authors used a combination of max pooling and convolutional layers to extract deep features. To relieve the overfitting problem, L2 regularization for the spectral convolutional neural network is adopted.

Mou et al. [33] proposed analyzing hyperspectral pixels to sequential data and using network reasoning to identify information categories. In this approach, a new activation function called parametric rectified tanh is created and used to analyze hyperspectral sequential data.

Nindam et al. [34] designed a new deep neural network architecture for classifying jasmine rice seed germination. In this architecture, the dataset of rice seed germination is collected and classified into three different classes: poor, good, and excellent germination.

Bensaoud and Kalita [35] proposed a new multitask learning framework to classify malware images. In this framework, malware features are extracted and used to create Portable Network Graphic (PNG) and bitmap images. Experimental results showed that the proposed model could detect a variety of obfuscation methods, such as encryption, instruction overlapping, and packing.

Based on the idea of replacing Inception modules with depthwise separable convolutions, a new deep convolutional network is proposed in [36]. Experimental results showed that the classification accuracy achieved by the new convolutional network is slightly higher than by Inception modules.

Zhong et al. [37] addressed the degradation problem of hyperspectral image classification accuracy in the deeper layers. To alleviate the influence of the classification accuracy degradation problem, the authors proposed adding identity mappings to the convolutional neural networks.

Based on the streamlined architecture, Howard et al. [38] proposed a new classification model used to generate lightweight deep neural networks. In this model, two global hyperparameters are created and utilized to achieve a trade-off between accuracy and latency.

In [39], the authors proposed a novel histopathology image recognition system to minimize the error rate and speed up breast cancer diagnosis. This work uses GoogLeNet to create a hybrid convolutional neural network, while hierarchy voting tactics and bagging techniques are adopted to improve classification performance.

Ghassemi et al. [40] addressed the difficulties that often tumor classification in MR images faces. In this work, the authors proposed a new deep-learning method. In this proposed method, the deep neural network is trained on different datasets of MR images, then used the trained network as a classifier to classify three tumor classes.

Xie et al. [41] proposed repeating building blocks to construct a new image classification modularized network called ResNeXt. The proposed network is multi-branch and homogeneous, in addition to containing three dimensions: depth, width, and cardinality. COCO detection set and ImageNet-5K are used to evaluate the performance of ResNeXt. Experimental results showed that ResNeXt achieved better classification accuracy than ResNet.

Ershad and Ramakrishnan [42] proposed a new two-stage approach for cervical cancer diagnosis in pap smear images. In this approach, the texture information of the cytoplasm and nucleolus is extracted. In this aspect, the author used a suitable threshold to segment the pap smear image, then classified the pap smear images with the optimized multi-layer feed-forward neural network. In this work, a genetic algorithm is used to optimize the classification accuracy of the proposed model. On the other hand, the cross-over process and innovative chromosomes are used to manage the parameters.

Attallah [43] proposed a new computer-aided diagnostic (CAD) model. In this model, the author proposed to extract features from multiple domains instead of only one domain. In this aspect, the author proposed to examine the effect of each set of handcrafted attributes on diagnostic accuracy, then used the principal component analysis to combine the whole deep learning features. Compared to other models, this model is less complex and more effective in retrieving several textural descriptors from different domains. However, this model can only be used for classifying pap smear images.

The ResNet model has been widely used in the literature. In the deeper layer of the ResNet model [2], the authors used Relu and shortcut connections to make connections between different layers to solve the exploding gradients problem [44]. However, when the dimensionalities between layers are different, projection shortcuts should be used for matching the dimensions, which inevitably leads over time to increasing the architecture complexity, maximizing the number of parameters, increasing the computational cost, and decreasing the classification accuracy, especially in the deeper branches of the network. A large number of parameters can increase the likelihood of the network being exposed to overfitting, especially when the sample size of the training set is relatively limited. On the other hand, utilizing Relu causes the vanishing gradient problem. In this case, the network will be unable to perform backpropagation, which hampers learning and convergence, and ultimately results in more accuracy degradation.

### Main Contributions

The main contributions of the proposed model can be summarized as follows:

1. The major problem of the Relu activation function is the deactivation of the non-positive numbers. The deactivation problem causes vanishing gradients, slower convergence, and degrading classification accuracy. Our proposed non-monotonic activation function (NMAF) succeeded in solving the deactivation of the non-positive numbers by activating the negative values and outputting small negative numbers instead of zero in Relu, which helped in enhancing the convergence speed and increasing the classification accuracy by 5%, 15%, and 5% for the non-noisy datasets, and 5%, 6%, and 21% for the noisy datasets.
2. To reduce the number of weights (parameters) and avoid stacking the outputs resulting from aggregating the values from layer to layer in our neural network, we created two effective techniques, 1×1 convolutions and symmetric factorization. Utilizing these two techniques contributed to reducing the parameter number by around 6 million parameters compared with ResNet50, which has helped reduce the run time of our network by 30 s per epoch.
3. After taking the essential information of the input image and the decrease in the complexity of the network into consideration, a balanced combination of residual network and inception blocks has been created and used to achieve an incredible classification accuracy of 90.20%, 78.20%, and 92.00% for non-noisy datasets, and 88.37%, 84.66%, and 75.00% for the noisy datasets.
4. To smartly manage underfitting and overfitting problems, appropriate parameters γ and α are created and used to control the slope of NMAF for negative and positive input values, respectively.

## 2. Proposed Neural Network

As mentioned above, in the ResNet model, the greater the depth of the network, the lower the classification accuracy, and the greater the training time and the number of parameters [15]. For that reason, in our proposed model, we avoid utilizing the deeper layers of the residual network. In our proposed model, instead of utilizing the deeper layers (deeper branches) of the ResNet, we have modified and used the inception blocks. On the other hand, we replaced the conventional Relu activation function with our proposed non-monotonic activation function (NMAF). In this aspect, the vanishing gradient problem has been addressed carefully in our non-monotonic activation function (NMAF). To smoothly avoid this problem, we proposed activating the negative values and outputting small negative numbers instead of zero in Relu. Thanks to adopting NMAF, our proposed model became able to expedite learning in the deeper layers during the training process, resulting in better classification accuracy, consuming less time, and utilizing fewer parameters.

Although our proposed method adopts the inception technique proposed in [28], our proposed model differs from [28] in many aspects. Firstly, in our proposed model, we adopt our proposed non-monotonic activation function (NMAF) instead of Relu. Secondly, the number of inception and reduction blocks is relatively different from that in [28]. Thirdly, the number of filters in each block and the sizes of each filter differ from [28]. Fourthly, in the classification layer, we apply an average pooling filter instead of global average pooling. Based on our experiments, we found that utilizing global average pooling significantly degrades classification accuracy. Fifthly, unlike [28], we did not find applying dropout essential in our network. Sixthly, in our neural network, we avoided adding an auxiliary classifier. We found that adding the auxiliary classifier causes instability during training, thus can decrease the classification accuracy as the number of classes increases.

In our neural network, we apply various filters with different sizes (1×1, 3×3, 5×5, and 7×7), which aligns with the fact that each image contains objects with different scales. Therefore, these objects must be processed through diverse sizes of filters [28].

In this work, we focus on increasing classification accuracy and reducing the training time and the number of parameters. To reach this goal, we proposed applying the adjusted residual layers to the shallower layers of our proposed network to capture more information from varying scales of the input images. In contrast, the modified inception blocks have been created and utilized in our deeper layers to avoid complexity and gain better classification accuracy with fewer parameters.

Our neural network uses 1×1 convolutions to reduce the dimension and the proposed on-monotonic activation function to activate the negative values and output small negative numbers. Adopting 1×1 convolutions followed by the proposed non-monotonic activation function (NMAF) allows not only to increase the number of blocks but also to maximize the size of each block without leading to any computational difficulties during the training process.

**Figure 1:**
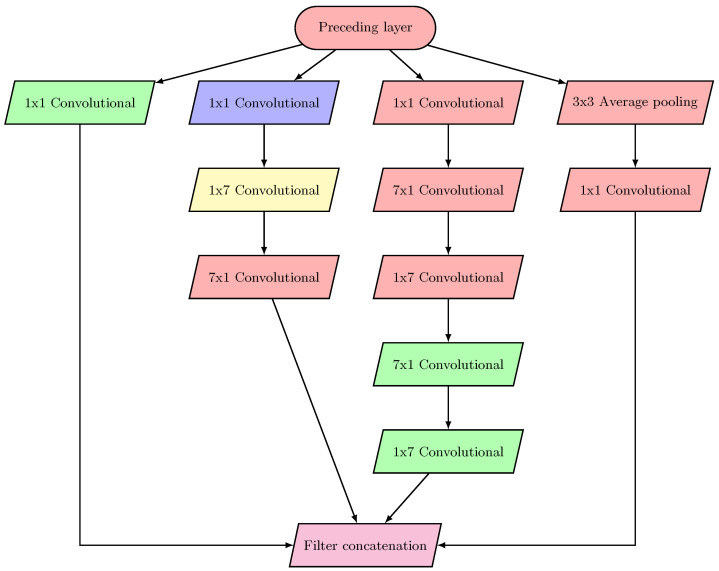
Block diagram of Inception II.

**Figure 2:**
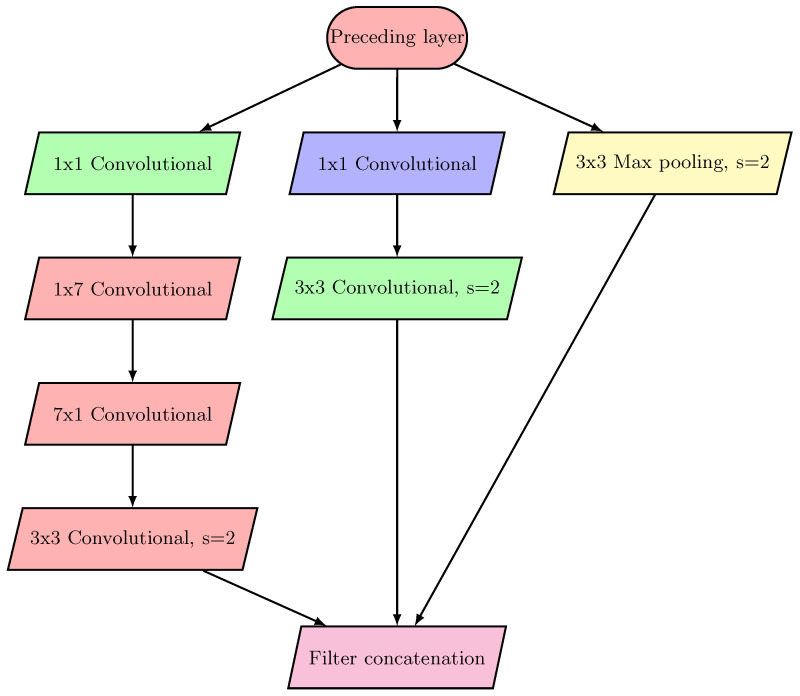
Block diagram of Reduction II.

**Figure A1:**
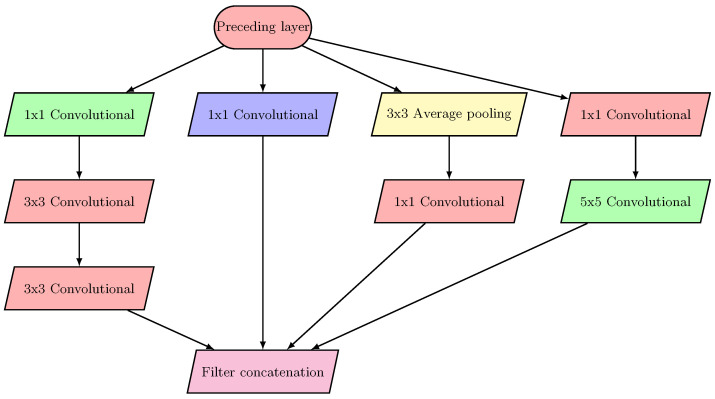
Block diagram of Inception I.

**Figure A2:**
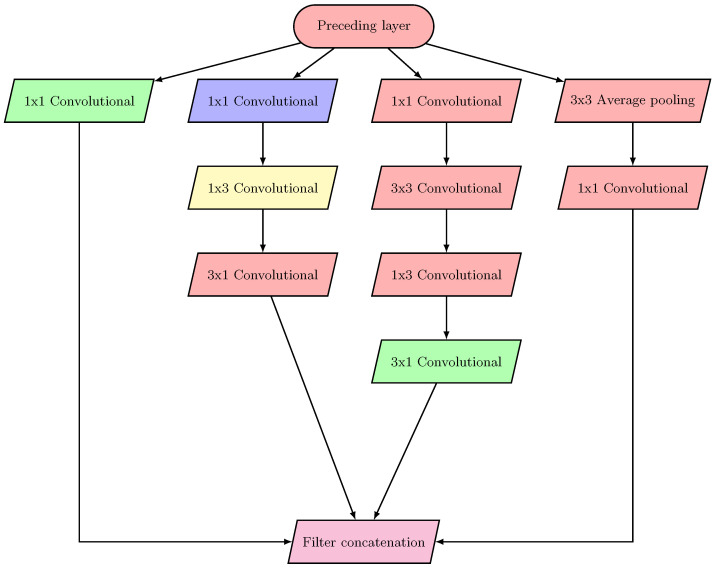
Block diagram of Inception III.

**Figure A3:**
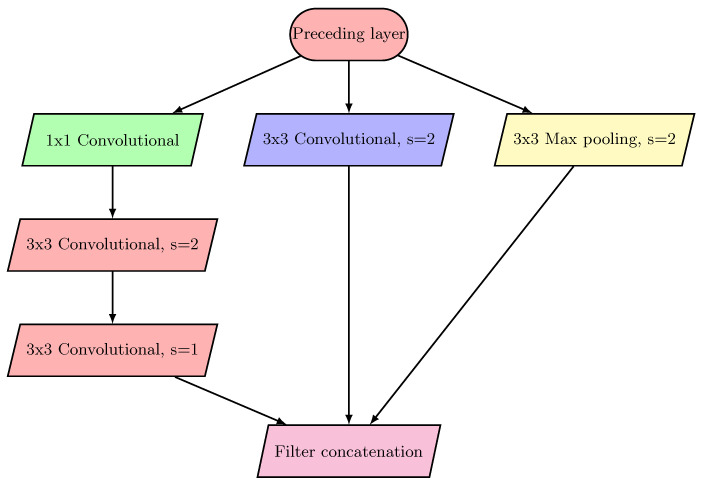
Block diagram of Reduction I.

For memory efficiency reasons, in our neural network architecture (Figure 1, Figure 2, Figure A1, Figure A2 and Figure A3), the filters with larger sizes are applied after 1×1 convolutions, while average-pooling and max-pooling are applied before 1×1 convolutions. This effective architecture enables us to avoid the output stacking problems generated by accumulating the values from layer to layer, thus preventing exploding gradients in the last layers of the network.

### 2.1. Inception and Reduction

In our network architecture, we modify and use the inception blocks to reduce the number of parameters, which results in less runtime and better accuracy.

Applying the inception modules in our model differs from [28] in six aspects:

1. Replacing the Relu activation function with the proposed non-monotonic activation function (NMAF).
2. The number of inception and reduction blocks differs from that in [28].
3. The number of filters and the size of each filter are different.
4. In the classification layer, the global average pooling filter is replaced with the average pooling filter.
5. Avoid utilizing dropout in our model.
6. Avert applying the auxiliary classifier.

As shown in Figure 1 and Figure 2, we utilize the symmetric factorization method to reduce the number of parameters in Inception block II and Reduction block II. In this method, first, we factorize the filter 7 × 7 into symmetric filters of sizes 1 × 7 and 7 × 1, then replace them with a series of 3 × 3 convolution filters. In this process, we reduce the number of parameters by 29%. Whereas, in Inception block III (Figure A2), we factorize the filter size of 3 × 3 into symmetric filters of sizes 1 × 3 and 3 × 1, which means that the number of parameters is reduced by 33%. In reduction block I (Figure A3), we first reduce the number of parameters by applying 1 × 1 convolution, then factorize the filter 5 × 5 that is received from Inception block I by 3 × 3 and 3 × 3 filters, which helps in reducing the number of parameters by 36%.

To calculate the number of parameters in each layer, let us suppose *w* is the shape of the filter’s width, *h* is the shape of the filter’s height, *m* is the number of filters in the previous layer, *n* is the number of filters in the current layer, and *b* is the bias, then the number of the parameters in the current layer can be calculated as follows:

parameters_number=(w∗h∗m+b)∗n

### 2.2. Proposed Network Architecture

**Figure 3:**
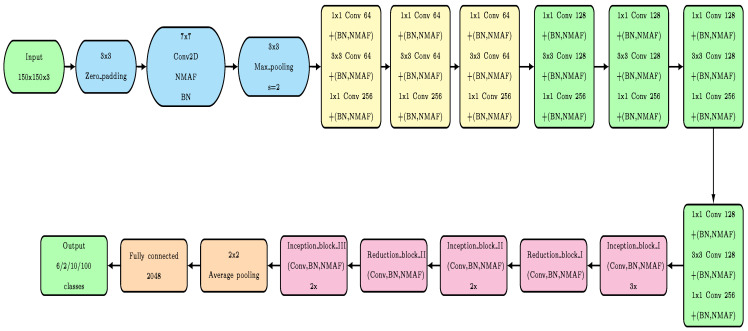
Block diagram of proposed neural network.

As shown in Figure 3, the architecture of our proposed network can be described as follows:

1. We start with the 3 × 3 zero padding to control the shrinkage of the input image dimensions.
2. We apply a convolution layer with 64 filters of size 7 × 7, batch normalization, and proposed non-monotonic activation function (NMAF).
3. Apply (3, 3) max pooling with a stride of (2, 2) to halve the parameters and computations.
4. Adopt three residual blocks (config: convolution layers with 64, 64, and 256 filters of sizes 1 × 1, 3 × 3, and 1×1, respectively). In these three blocks, each convolution layer is followed by batch normalization and NMAF.
5. Apply four residual blocks (config: convolution layers with 128, 128, and 256 filters of sizes 1×1, 3×3, and 1×1, respectively).
6. In this phase, we apply three inception blocks (config: convolution layers, batch normalization, and our proposed non-monotonic Activation Function (NMAF)), where Inception block I is repeated three times, while Inception block II and Inception block III are repeated twice.
7. Two reduction blocks (config: convolution layers, batch normalization, and NMAF) are applied.
8. In the classification layer, we apply average pooling with a stride of (2, 2).
9. Adopting a fully connected layer with 2048 neurons.
10. Getting the final output layer with 100, 10, and 6 classes.

### 2.3. Proposed Non-Monotonic Activation Function (NMAF)

The activation function has a pivotal influence on the runtime complexity and training accuracy. For that reason, the activation function represents the cornerstone of neural networks. The most common example of activation functions is rectified linear activation function (Relu), which is extensively used in the literature. In ResNet [2] and Inception [28] models, the authors used Relu in their networks. In this activation function, all outputs of negative inputs are arbitrarily forced to zero, which leads to the deactivation of many neurons during training. The deactivation problem causes damage to the neural network capability, which results in a vanishing gradient, slower convergence, and more accuracy degradation [45]. To overcome these problems, we proposed a non-monotonicity activation function called NMAF. Our proposed activation function (NMAF) addresses the problem of deactivating the non-positive numbers by activating the negative values and outputting small negative numbers instead of zero in Relu, thus enhancing the convergence speed. It is noteworthy that our proposed activation function (NMAF) gains its non-monotonicity feature from the negative part of its graph.

Experimental results provide solid evidence that NMAF can adapt to various datasets and achieve a significant improvement in learning both positive and negative values compared to Relu, which enhances our model’s classification performance. NMAF also has a better capability in training deeper networks than Relu.

In our proposed non-monotonic activation function (NMAF), γ is used to control the slope of NMAF for negative input values. In contrast, α is used to manage the slope of NMAF for positive input values. In this aspect, we conducted our experiments with the parameter γ in the range 0<γ<1. Based on our experiments, we found that adopting γ with a value greater than one usually leads to an exploding gradient problem. In contrast, adopting γ with a value of less than zero always results in a vanishing negative values problem.

**Figure A4:**
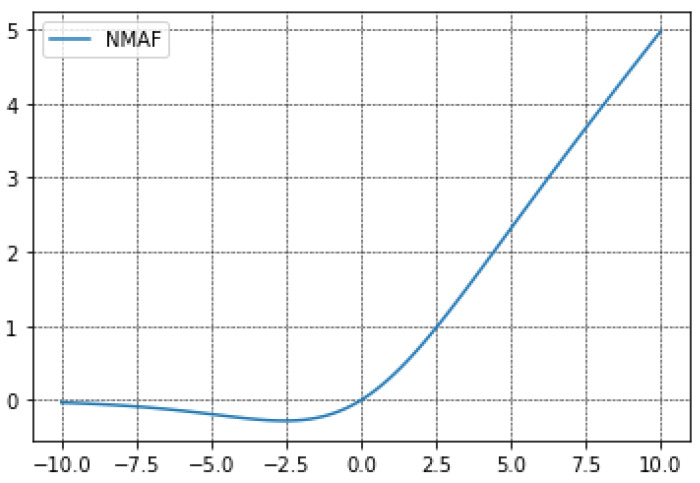
Graph of the proposed non-monotonic activation function (NMAF).

Our proposed non-monotonic activation function (NMAF) is visualized in Figure A4.

The equation that represents our proposed non-monotonic activation function (NMAF) is as follows:

(1) $$f\left(x\right)=x*sin\left(\alpha \right)*\sigma \left(\frac{2x}{\gamma +1}\right)=\frac{x*sin(\alpha )}{1+exp(\frac{-2x}{\gamma +1})},$$

where

(2) $$\sigma \left(\frac{2x}{\gamma +1}\right)=sigmoid\left(\frac{2x}{\gamma +1}\right)=\frac{1}{1+exp(\frac{-2x}{1+\gamma })}$$

The derivative of NMAF can be calculated as follows:

$$\begin{array}{cc} f^{\prime }\left(x\right) & =\frac{sin\left(\alpha \right)\left(1+exp\left(\frac{-2x}{\gamma +1}\right)\right)+\frac{2}{\gamma +1}exp\left(\frac{-2x}{\gamma +1}\right)\left(x*sin\left(\alpha \right)\right)}{{\left(1+exp\left(-x\right)\right)}^{2}}\\ \; & =sin\left(\alpha \right)\left[\frac{\left(1+exp\left(\frac{-2x}{\gamma +1}\right)\right)+\frac{2x}{1+\gamma }*exp\left(\frac{-2x}{\gamma +1}\right)}{{\left(1+exp\left(\frac{-2x}{\gamma +1}\right)\right)}^{2}}\right]\\ \; & =sin\left(\alpha \right)[\sigma \left(\frac{2x}{\gamma +1}\right)+\frac{2x}{\gamma +1}\left(\sigma \left(\frac{2x}{\gamma +1}\right)-\sigma {\left(\frac{2x}{\gamma +1}\right)}^{2}\right)]\\ \; & =\frac{2}{\gamma +1}*f\left(x\right)+sin\left(\alpha \right)*\sigma \left(\frac{2x}{\gamma +1}\right)-\frac{2}{\gamma +1}*f\left(x\right)*\sigma \left(\frac{2x}{\gamma +1}\right)\\ \; & =\frac{2}{\gamma +1}*f\left(x\right)+\sigma \left(\frac{2x}{\gamma +1}\right)\left[sin\left(\alpha \right)-\frac{2}{\gamma +1}*f\left(x\right)\right] \end{array}$$

## 3. Experimental Results

In our experiments, network training and weights initialization have been completed from scratch. In these experiments, we use the extended version of stochastic gradient descent (Adam optimizer) with a mini-batch size of 64 and a learning rate of 0.001 with a lower bound of 0.000001, in which the learning rate reduces by a factor of 0.3. In this aspect, extensive experiments have been conducted on Intel image classification, CIFAR-10, and 100 Sports image classification datasets to evaluate the performance of our proposed model. As shown in Table 1, the images in these datasets are classified into 6, 10, and 100 classes, respectively. In these three datasets, our proposed model is trained on 14,034, 50,000, and 13,572 training images, evaluated on 7301, 5000, and 500 validation images, and tested on 3000, 5000, and 500 testing images. We use these datasets to provide robust evidence that our proposed network has a significant ability to improve the classification results, regardless of the datasets’ features. On the other hand, the comparisons in this section are conducted to verify the effectiveness of utilizing NMAF and activating the negative inputs on the classification performance of our proposed model.

**Figure 4:**
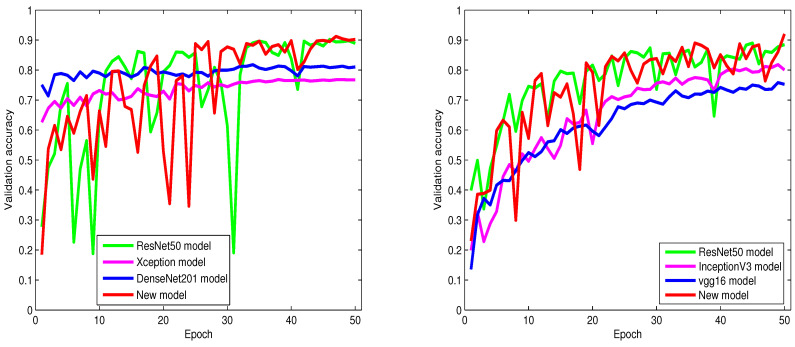
From left to right: the validation accuracy of the trained models on the Intel Image Classification and CIFAR-10 datasets.

**Figure 5:**
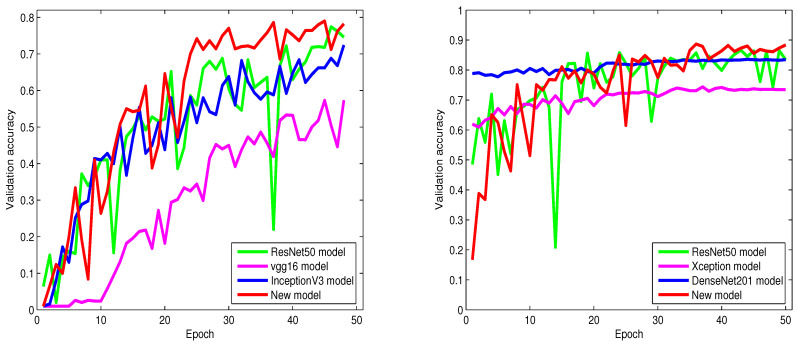
From left to right: the validation accuracy of the trained models on the 100 Sports Image Classification dataset and noisy (Gaussian noise) Intel Image Classification dataset.

**Figure 6:**
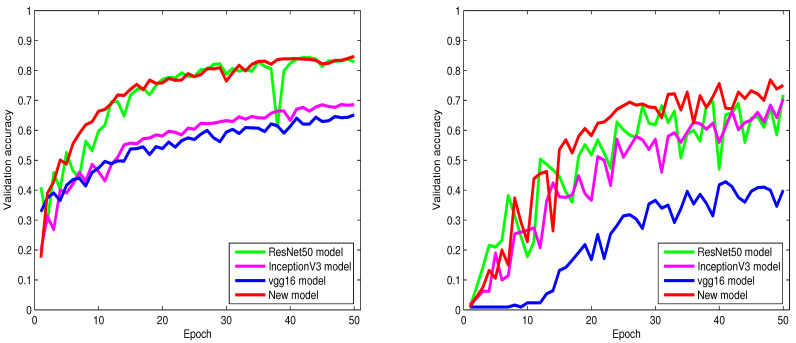
From left to right: the validation accuracy of the trained models on the noisy (Gaussian noise) CIFAR-10 dataset and noisy (Gaussian noise) 100 Sports image classification dataset.

From Table 2, Table 3, Table 4, Table 5, Table 6 and Table 7 and Figure 4, Figure 5 and Figure 6, we can notice that the classification accuracy of the six models differs with databases. However, in most cases, our proposed model consistently offers the highest classification accuracy among all models, which provides strong evidence that adopting the modified inception technique and NMAF in our model contributed to making significant enhancements to the classification performance. The information in Table 2, Table 3, Table 4, Table 5, Table 6 and Table 7 and Figure 4, Figure 5 and Figure 6 also provide strong evidence supporting the fact that our proposed network is not only effective for a particular dataset but is also applicable to different datasets. From these tables, we can also infer that our proposed algorithm consistently outperforms the state-of-the-art classification algorithms by a large margin.

From Table 2 and Table 3, it is obvious to see that the Xception, VGG16, InceptionV3, and DenseNet201 models distinctly underperform compared to ResNet50. Contrary to these four models, our proposed model shows improved performance and achieves high classification accuracy with a remarkable boost of 1.3% and 4.6% over the ResNet50 model. On the other hand, as shown in Table 2, our proposed model achieves 1.3%, 13.5%, and 9.2% higher accuracy over the ResNet50, Xception, and DenseNet201 models, respectively.

The runtime of ResNet and our proposed models are detailed in Table 8, Table 9 and Table 10. These tables show that the training procedure of the ResNet model is notably more time-consuming than our proposed model. From Table 9, we can observe that the runtime of the ResNet50 model is longer by about 23 min compared to our proposed model.

To show the extent of the influence of the noise on the classification accuracy of the six models, we propose to add Gaussian noise to the Intel images classification dataset, CIFAR-10 dataset, and 100 Sports image classification dataset. As described in Table 5, Table 6 and Table 7, our proposed model shows to be adaptable and accomplishes brilliant classification accuracy with challenging noisy datasets and a large number of classes. On the other hand, the results in these tables give solid evidence that the classification performance of the proposed algorithm is not affected deeply by real-world natural influences such as noise. From these tables, we can observe that our proposed model achieves pleasant classification accuracy of 88.37%, 84.66%, and 75% and outperforms the state-of-the-art models.

As shown in Table 6 and Table 7, ResNet50 offers a higher classification accuracy than Inception V3 and Vgg16 models. Nevertheless, ResNet50 classification accuracy is less impressive compared with our proposed model.

In Table 5, our proposed model produces impressive classification accuracy of 5%, 15%, and 5% over ResNet50, Xception, and DenseNet201 models, respectively.

Based on Table 2, Table 3 and Table 4, our proposed model yields classification accuracy of 0.9020, 0.7820, and 0.9200 for Intel images classification, 100 sports image classification, and CIFAR-10 datasets, respectively. From these results, we deduce that adopting the proposed non-monotonic activation function (NMAF) enables our network to adapt and change smoothly in various datasets and significantly enhances classification accuracy. These tables also report the number of parameters (weights) of six models. The main observation in these tables is that the numbers of parameters of Xception [36], DenseNet201 [22], and Vgg16 [20] models are so high compared with ResNet50 [2]. Nevertheless, the number of parameters of ResNet50 is still higher by a large margin (about 6M weights) compared with our proposed model. It is noteworthy that increasing the number of weights maximizes the likelihood of the network being exposed to overfitting, especially when the sample size of the training set is relatively limited.

As can be seen in Figure 3, the residual network part of our neural network contains three 3-layer blocks with 64 and 256 filters and four 3-layer blocks with 128 and 256 filters. Each convolution layer in these blocks is followed by batch normalization and our proposed non-monotonic activation function (NMAF), respectively. In contrast, in the inception network part, we repeat Inception block I three times and two times for Inception blocks II and III, while both reduction blocks I and II are applied only once.

**Figure A5:**
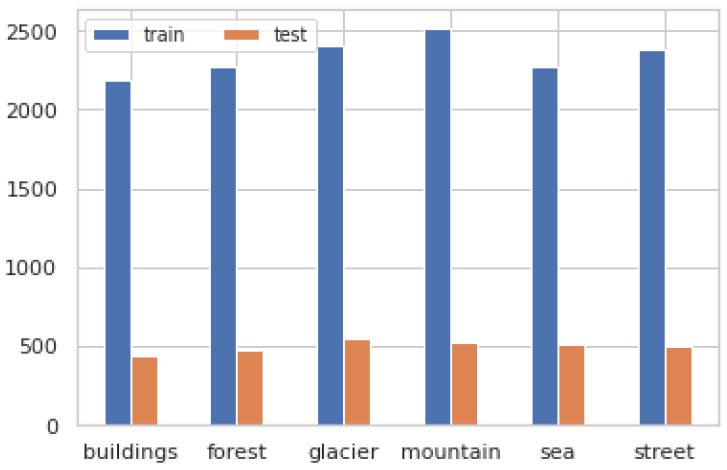
Training and testing labels visualization of Intel image classification dataset.

**Figure A6:**
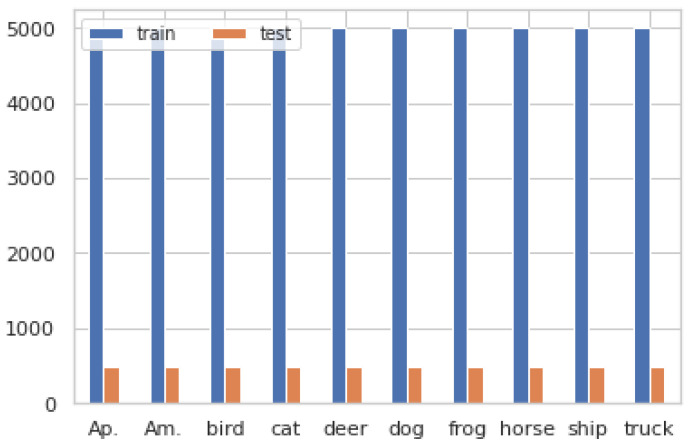
Training and testing labels (airplane, automobile, bird, cat, deer, dog, frog, horse, ship, truck) visualization of CIFAR-10 dataset.

Training and testing labels visualization of the Intel image classification and CIFAR-10 datasets are depicted in Figure A5 and Figure A6.

**Figure A7:**
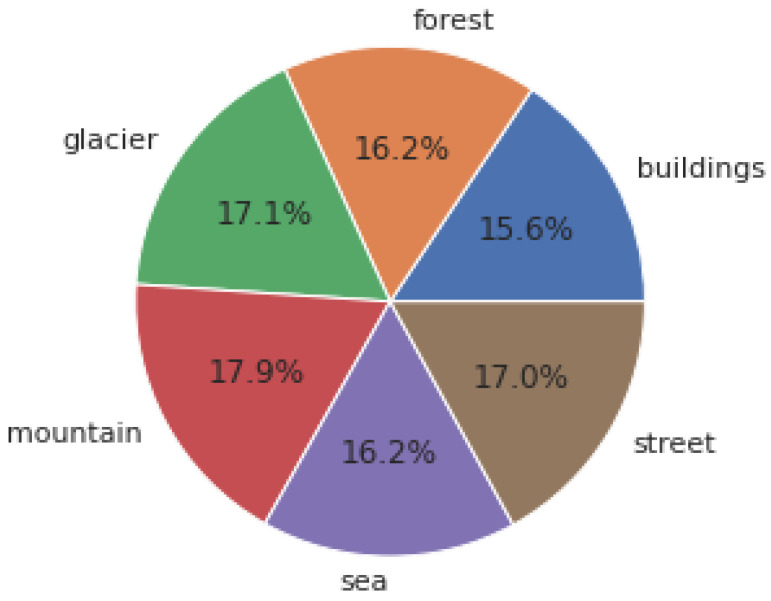
Percentage of each class of Intel image classification dataset.

The percentage of each class of the Intel image classification dataset is displayed in Figure A7.

Figure 1, Figure 2, Figure A1, Figure A2 and Figure A3 depict the block diagrams of Inception blocks I-III and Reduction blocks I and II.

**Figure A8:**
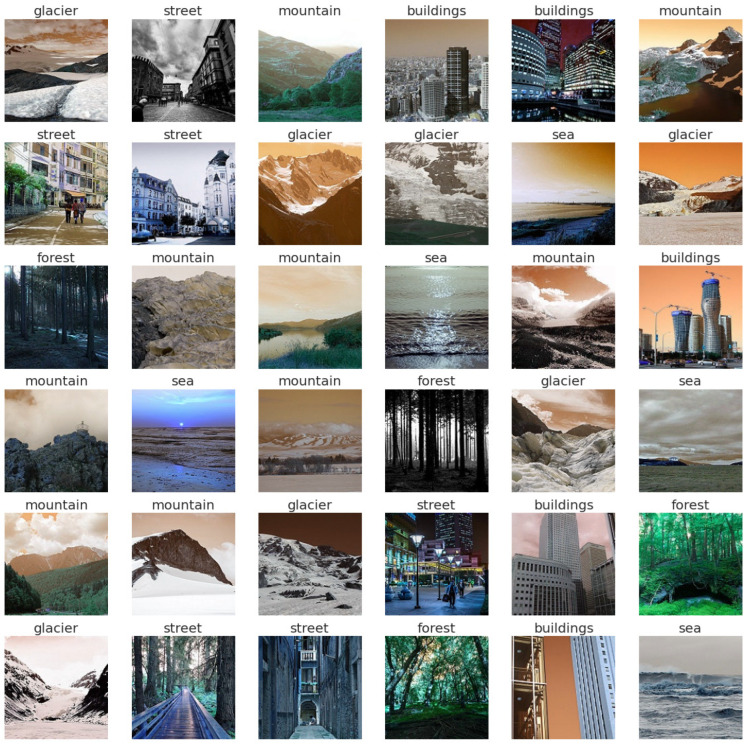
Random predicted images with their labels predicted by our proposed model.

Figure A8 shows 36 random predicted images (from the Intel classification images dataset) plus their predicted labels produced by applying our proposed model.

Figure 4, Figure 5 and Figure 6 show the behaviors of six different models. From these figures, we can discover that our proposed algorithm converges faster than other models. As shown in Figure 4 (left), from epoch zero to epoch 25, our proposed algorithm sometimes shows slightly lower accuracy than ResNet50. However, from epoch 25 and above, our proposed algorithm gradually achieves higher classification accuracy and convergence than ResNet50.

**Figure 7:**
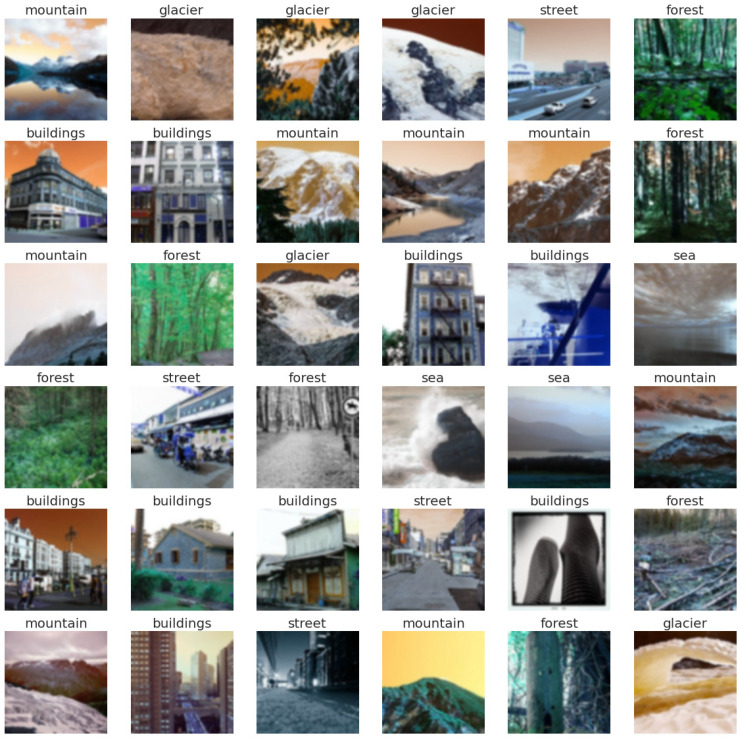
Noisy random predicted images with their labels predicted by our proposed model.

Figure 7 depicts 36 noisy random predicted images plus their predicted labels produced by applying our proposed model. As shown in this figure, most of the images’ features are corrupted by noise, which makes the classification mission quite hard. However, all images in this figure are correctly classified, except the first image (glacier image) in the third row is misclassified as a mountain. We believe that the main reason for misclassification is due to the close similarity between the features of mountain and glacier images.

Table 11 shows the validation accuracy of five different models. In this table, our proposed model achieved the best result among all models.

Table 12 shows the result of our proposed model with the Gaussian linear error unit (GeLU) activation function, exponential linear unit ELU, and our proposed activation function (NMAF). From this table, we can see that our proposed model with NMAF achieved better results with less runtime compared with New-Elu and New-Gelu.

From the above results and discussions, we can say that our experiments demonstrated that our proposed convolutional neural network performs image classification with better efficiency than the ResNet classification model. In addition to being less time-consuming, it also contains fewer parameters.

Based on our experimental results, the CIFAR-10 dataset is more time-consuming than Intel image classification and 100 sports image classification datasets. For the CIFAR-10 dataset, the most time-consuming epoch is the first epoch. This epoch consumes 166 s. In contrast, the less time-consuming epoch is epoch number 27, which only consumes 141 s. For the Intel image classification dataset, the first epoch is the most time-consuming, which consumes 102 s, while the less time-consuming one is epoch number 38, which only consumes 82 s. In the 100 sports image classification dataset, epoch one is the most time-consuming epoch, which consumes 100 s, whereas the less time-consuming epoch is the 12th one, which only consumes 78 s.

For the CIFAR-10 dataset, there are 782 steps for each epoch. In the first epoch, each step consumes 191 ms, and 180 ms for each step in epoch 27. For the Intel image classification dataset, there are 220 steps per epoch. In the first epoch, each step takes 388 ms, and 374 ms for each step in epoch number 38. For the 100 sports image classification dataset, there are 213 steps per epoch. In the first epoch, each step takes 390 ms, and 365 ms per step in epoch 12.

## 4. Conclusions

In this paper, accuracy degradation, time complexity, and increasing the used weights (parameters) during the training process have been addressed carefully. By replacing the deeper branches of the residual networks with the adjusted inception blocks and the Relu activation function with our proposed non-monotonic activation function (NMAF), our proposed model managed to decrease the number of training parameters, improve training stability, save more time, and gain better classification accuracy. Utilizing fewer parameters reduces the likelihood of the network being exposed to overfitting and improves the rate of convergence. In our proposed non-monotonic activation function (NMAF), the negative part gives the NMAF non-monotonicity property, activates the negative values, and outputs small negative numbers instead of zero in Relu. In this case, the network continues to process the negative inputs, and we use γ to control the saturation degree of the negative inputs. To decrease the number of weights and prevent exploding gradients, symmetric factorization, and 1×1 convolutions are created and utilized to avoid stacking the outputs resulting from aggregating the values from layer to layer. Based on experimental results, our proposed algorithm shows a significant ability to adapt and change over different datasets and achieve impressive enhancements in classifying clean and noisy datasets. To evaluate the performance of our proposed model on the noisy datasets, we propose adding an additive Gaussian noise to Intel image classification, CIFAR, and 100 Sports Image Classification datasets. Our proposed model achieves state-of-the-art results, with classification accuracies of 88.37%, 84.66%, and 75.00%. Moreover, our proposed model produces remarkable results with accuracies of 90.20%, 78.20%, and 92.00% for the same non-noisy datasets. When classifying the noisy datasets, our proposed model performs impressively, achieving classification accuracy of 5%, 15%, and 5% over other models, while achieving accuracies of 21%, 6%, and 5% for the non-noisy datasets. In addition to the accuracy gains, our proposed model has successfully reduced the number of parameters by 6 million compared to the ResNet50 model. We believe that our proposed method is inclusive and generalizable to other types of classification. In future work, we will address how to overcome the slow convergence in the starting epochs and manage the dependency on batch normalization. To achieve better classification accuracy, we will consider taking the learnable parameter (γ) into account.

## Figures and Tables

**Table 1:** Details of the utilized datasets in six different models.

Datasets	Classes No.	Training	Test	Validation
Intel Image	6	14,034	3000	7301
CIFAR-10	10	50,000	5000	5000
100 Sport	100	13,572	500	500

**Table 2:** Validation accuracy and the number of parameters of Intel images classification dataset performed with four different classification models.

Model	Parameters	Validation Accuracy
ResNet50 model	23,636,870	0.8893
Xception model	73,301,550	0.7673
DenseNet201 model	49,790,534	0.8103
New model	17,665,670	0.9020

**Table 3:** Validation accuracy and the number of parameters of 100 Sport classification images performed with four different classification models.

Model	Parameters	Validation Accuracy
ResNet50 model	24,407,012	0.7360
VGG16 model	65,464,228	0.5731
InceptionV3 model	20,234,180	0.7240
New model	18,435,812	0.7820

**Table 4:** Validation accuracy and the number of parameters of CIFAR-10 images dataset performed with four different classification models.

Model	Parameters	Validation Accuracy
ResNet50 model	23,608,202	0.8848
InceptionV3 model	20,049,770	0.8006
VGG16 model	50,415,434	0.7526
New model	17,637,002	0.9200

**Table 5:** Validation accuracy and the validation loss of noisy Intel image classification dataset performed with four different classification models.

Model	Validation Loss	Validation Accuracy
ResNet50 model	0.5039	0.8343
Xception model	0.7155	0.7343
DenseNet201 model	0.4746	0.8347
New model	0.3144	0.8837

**Table 6:** Validation accuracy and the validation loss of noisy CIFAR-10 dataset performed with four different classification models.

Model	Validation Loss	Validation Accuracy
ResNet50 model	0.5358	0.8294
Inception V3 model	0.9051	0.6852
VGG16 model	1.0064	0.6514
New model	0.4754	0.8466

**Table 7:** Validation accuracy and the validation loss of noisy 100 Sports Image Classification dataset performed with four different classification models.

Model	Validation Loss	Validation Accuracy
ResNet50 model	1.0969	0.7180
Inception V3 model	1.0712	0.7040
VGG16 model	2.2648	0.4000
New model	0.9549	0.7500

**Table 8:** Computational cost of the ResNet50 model and proposed model for the 100 sports image classification dataset.

Model	Total Time	Time/Epoch	Time/Tep
New model	3967 s	79 s	368 ms
ResNet50	4012 s	80 s	380 ms

**Table 9:** Computational cost of the ResNet50 model and proposed model for the Intel image classification dataset.

Model	Total Time	Time/Epoch	Time/Tep
New model	4210 s	84 s	380 ms
ResNet50	5563 s	113 s	500 ms

**Table 10:** Computational cost of the ResNet50 model and proposed model for the CIFAR-10 dataset.

Model	Total Time	Time/Epoch	Time/Tep
New model	7239 s	145 s	185 ms
ResNet50	7594 s	152 s	195 ms

**Table 11:** Validation accuracy of CIFAR-10 dataset performed with five different classification models.

Model	[45]	[46]	[47]	[48]	New
Val_accuracy	89.28%	91.10%	71.66%	87.57%	92.00%

**Table 12:** Validation accuracy and run time of Intel images classification dataset performed with our proposed model with ELU, GELU, and our NMAF activation functions.

Model	Time/Epoch	Validation Acccuracy
New-ELU	99 s	0.9012
New-GELU	108 s	0.8957
New-NMAF	84 s	0.9020

## Data Availability

The datasets used in this work can be found in the links below: Intel Image Classification dataset: https://www.kaggle.com/puneet6060/intel-image-classification/ (accessed date: 29 January 2023). CIFAR-10 dataset: https://www.kaggle.com/datasets/ayush1220/cifar10/ (accessed date: 29 January 2023). 100 Sports Image Classification dataset: https://www.kaggle.com/datasets/gpiosenka/sports-classification (accessed date: 29 January 2023).

## Abbreviations

The following abbreviations are used in this manuscript:

ResNet	Residual neural network
NMAF	Non-monotonic activation function
DenseNet	Dense convolutional network

## References

[1] Lei Z., Duan P., Hong X., Mota J.F.C., Shi J., Wang C.-X. (2022). Progressive deep Image compression for hybrid contexts of image classification and reconstruction. IEEE J. Sel. Areas Commun..

[2] He K., Zhang X., Ren S., Sun J. Deep residual learning for image recognition. Proceedings of the IEEE Conference on Computer Vision and Pattern Recognition (CVPR).

[3] Bengio Y., Simard P., Frasconi P. (1994). Learning long-term dependencies with gradient descent is difficult. IEEE Trans. Neural Netw..

[4] Girshick R. Fast R-CNN. Proceedings of the IEEE International Conference on Computer Vision.

[5] Shin H.C., Roth H.R., Gao M., Xu L.L.Z., Nogues I., Yao J., Mollura D., Summers R.M. (2016). Deep convolutional neural networks for computer-aided detection: CNN architectures, dataset characteristics and transfer learning. IEEE Trans. Med. Imaging.

[6] Selvaraju R.R., Cogswell M., Das A., Vedantam R., Parikh D., Batra D. Ablation-CAM: Visual explanations for deep convolutional network via gradient-free localization. Proceedings of the IEEE International Conference on Computer Vision.

[7] Yahya A.A., Tan J., Hu M. (2021). A Novel handwritten digit classification system based on convolutional neural network approach. Sensors.

[8] He K., Zhang X., Ren S., Sun J. Identity mappings in deep residual networks. Proceedings of the European Conference on Computer Vision.

[9] Zhang X., Saniie J. Reinforcement learning based neural architecture search for flaw detection in intelligent ultrasonic imaging NDE system. Proceedings of the IEEE International Ultrasonics Symposium.

[10] Tajbakhsh N., Shin J.Y., Gurudu S.R., Hurst R.T., Kendall C.B., Gotway M.B., Liang J. (2016). Convolutional neural networks for medical image analysis: Full training or fine tuning?. IEEE Trans. Med. Imaging.

[11] Lange S., Ulbrich F., Goehring D. Online vehicle detection using deep neural networks and lidar based preselected image patches. Proceedings of the IEEE Intelligent Vehicles Symposium.

[12] Surya T., Chitra Selvi S., Selvaperumal S. (2022). The IoT-based real-time image processing for animal recognition and classification using deep convolutional neural network (DCNN). Microprocess. Microsyst..

[13] Rasheed A., Umar A.I., Shirazi S.H., Khan Z., Nawaz S., Shahzad M. (2022). Automatic eczema classification in clinical images based on hybrid deep neural network. Comput. Biol. Med..

[14] Hafiz R., Haque M.R., Rakshit A., Uddin M.S. (2022). Image-based soft drink type classification and dietary assessment system using deep convolutional neural network with transfer learning. J. King Saud Univ.-Comput. Inf. Sci..

[15] Han L., Yu C., Xiao K., Zhao X. (2019). Anew method of mixed gas identification based on a convolutional neural network for time series classification. Sensors.

[16] Glorot X., Bengio Y. Understanding the difficulty of training deep feedforward neural networks. Proceedings of the Thirteenth International Conference on Artificial Intelligence and Statistics, Chia Laguna Resort.

[17] Calık R.C., Demirci M.F. Cifar-10 image classification with convolutional neural networks for embedded systems. Proceedings of the IEEE/ACS 15th International Conference on Computer Systems and Applications (AICCSA).

[18] Peng S., Huang H., Chen W., Zhang L., Fang W. (2022). More trainable inception-ResNet for face recognition. Neurocomputing.

[19] Lin1 M., Chen Q., Yan S. (2013). Network In Network. arXiv.

[20] Simonyan K., Zisserman A. (2014). Very deep convolutional networks for large-scale image recognition. arXiv.

[21] Krizhevsky A., Sutskever I., Hinton G.E. (2017). ImageNet classification with deep convolutional neural networks. Commun. ACM.

[22] Huang G., Liu Z., Maaten L.V.D., Weinberger K.Q. Densely connected convolutional networks. Proceedings of the IEEE Conference on Computer Vision and Pattern Recognition.

[23] Ikechukwu A.V., Murali S., Deepu R., Shivamurthy R.C. (2021). ResNet-50 vs VGG-19 vs training from scratch: A comparative analysis of the segmentation and classification of Pneumonia from chest X-ray images. Glob. Trans. Proc..

[24] Cheng J., Tian S., Yu L., Gao C., Kang X., Ma X., Wu W., Liu S., Lu H. (2022). ResGANet: Residual group attention network for medical image classification and segmentation. Med. Image Anal..

[25] Sarwinda D., Paradisa R.H., Bustamam A., Anggia P. Deep learning in image classification using residual network (ResNet) variants for detection of colorectal cancer. Proceedings of the 5th International Conference on Computer Science and Computational Intelligence.

[26] Zoph B., Vasudevan V., Shlens J., Le Q.V. Learning transferable architectures for scalable image recognition. Proceedings of the IEEE/CVF Conference on Computer Vision and Pattern Recognition.

[27] Zoph B., Le Q.V. Neural architecture search with reinforcement learning. Proceedings of the International Conference on Learning Representations.

[28] Szegedy C., Liu W., Jia Y., Sermanet P., Reed S., Anguelov D., Erhan D., Vanhoucke V., Rabinovich A. Going deeper with convolutions. Proceedings of the IEEE Conference on Computer Vision and Pattern Recognition (CVPR).

[29] Szegedy C., Vanhoucke V., Ioffe S., Shlens J., Wojna Z. Rethinking the inception architecture for computer vision. Proceedings of the IEEE Conference on Computer Vision and Pattern Recognition.

[30] Szegedy C., Ioffe S., Vanhoucke V., Alemi A.A. Inception-v4, inception-resnet and the impact of residual connections on learning. Proceedings of the Thirty-First AAAI Conference on Artificial Intelligence.

[31] Clevert D.-A., Unterthiner T., Hochreiter S. Fast and accurate deep network learning by exponential linear units (elus). Proceedings of the 4th International Conference on Learning Representations.

[32] Chen Y., Jiang H., Li C., Jia X., Ghamisi P. (2016). Deep feature extraction and classification of hyperspectral images based on convolutional neural networks. IEEE Trans. Geosci. Remote Sens..

[33] Mou L., Ghamisi P., Zhu X.X. (2017). Deep recurrent neural networks for hyperspectral image classification. IEEE Trans. Geosci. Remote Sens..

[34] Nindam S., Manmai T., Lee H.J. Multi-label classification of Jasmine Rice germination using deep neural network. Proceedings of the 7th International Conference on Business and Industrial Research.

[35] Bensaoud A., Kalita J. (2022). Deep multi-task learning for malware image classification. J. Inf. Secur. Appl..

[36] Chollet F. Xception: Deep learning with depthwise separable convolutions. Proceedings of the IEEE Conference on Computer Vision and Pattern Recognition.

[37] Zhong Z., Li J., Ma L., Jiang H., Zhao H. Deep residual networks for hyperspectral image classification. Proceedings of the IEEE International Geoscience and Remote Sensing Symposium.

[38] Howard A.G., Zhu M., Chen B., Kalenichenko D., Wang W., Weyand T., Andreetto M., Adam H. (2017). MobileNets: Efficient convolutional neural networks for mobile vision applications. arXiv.

[39] Guo Y., Dong H., Song F., Zhu C., Liu J. Breast cancer histology image classification based on deep neural networks. Proceedings of the International Conference Image Analysis and Recognition.

[40] Ghassemi N., Shoeibi A., Rouhani M. (2020). Deep neural network with generative adversarial networks pre-training for brain tumor classification based on MR images. Biomed. Signal Process. Control..

[41] Xie S., Girshick R., Dollár P., Tu Z., He K. Aggregated residual transformations for deep neural networks. Proceedings of the IEEE Conference on Computer Vision and Pattern Recognition.

[42] Ershad S.F., Ramakrishnan S. (2022). Cervical cancer diagnosis based on modified uniform local ternary patterns and feed forward multilayer network optimized by genetic algorithm. Comput. Biol. Med..

[43] Attallah O. (2023). Cervical cancer diagnosis based on multi-domain features using deep learning enhanced by handcrafted descriptors. Appl. Sci..

[44] Wen L., Li X., Gao L. (2020). A transfer convolutional neural network for fault diagnosis based on ResNet-50. Neural Comput. Appl..

[45] Maniatopoulos A., Mitianoudis N. (2021). Learnable leaky relu (LeLeLU): An alternative accuracy-optimized activation function. Information.

[46] Ho-Phuoc T. (2019). CIFAR10 to compare visual recognition performance between deep neural networks and humans. arXiv.

[47] Doon R., Rawat T.K., Gautam S. Cifar-10 classification using deep convolutional neural network. Proceedings of the IEEE Punecon.

[48] Ramachandran P., Zoph B., Le Q.V. Searching for activation functions. Proceedings of the ICLR 2018 Conference Blind Submission.

